# The intensity of joint pain in relation to changes in serum TNFα during therapy with anti-TNFα inhibitors

**DOI:** 10.1007/s10787-019-00564-x

**Published:** 2019-01-24

**Authors:** Dorota Sikorska, Edyta Kawka, Rafał Rutkowski, Włodzimierz Samborski, Janusz Witowski

**Affiliations:** 1 Department of Rheumatology and Rehabilitation, Poznan University of Medical Sciences, 28-Czerwca 1956 Street 135/147, 61-545 Poznan, Poland; 2 Department of Pathophysiology, Poznan University of Medical Sciences, Poznan, Poland

**Keywords:** Tumor necrosis factor-alpha, Rheumatoid arthritis, Biological disease-modifying antirheumatic drug

## Abstract

**Introduction:**

Tumor necrosis factor-alpha (TNFα) inhibitors have significantly improved the outcomes of treatment for rheumatoid arthritis (RA). In the present study, we aimed to determine whether serum levels of TNFα during therapy with TNFα inhibitors do really reflect the disease activity and correspond to the intensity of pain experienced.

**Materials and methods:**

Thirty RA patients were examined before and after 12 weeks of routine therapy with TNFα inhibitors. Serum levels of TNFα were measured with a high-sensitivity immunoassay and related to patients’ clinical and biochemical status. Disease activity was assessed by the modified disease activity score (DAS28).

**Results:**

A median relative change in TNFα was 13%. The patients were stratified according to whether the relative change in serum TNFα after therapy was above or below this median value. The patients from both subgroups did not differ in baseline characteristics and response to therapy. However, the patients in whom serum TNFα increased after therapy above the median value had more tender joints after treatment than patients from the other group. Consequently, the number of tender joints after the treatment correlated with absolute TNFα concentrations at this time (*r* = 0.37; *p* = 0.049) and the magnitude of changes in serum TNFα correlated with a change in the number of tender joints (*r* = − 0.48; *p* = 0.008).

**Conclusions:**

Circulating TNFα levels did not decrease in RA patients treated with TNFα inhibitors, despite clinical and biochemical improvement. It is possible, that circulating TNFα is responsible for the persistence of joint pain in this group of patients.

## Introduction

Tumor necrosis factor-alpha (TNFα) is thought to contribute critically to joint destruction in RA (Furst and Emery 2014). TNFα activates leukocytes and synovial fibroblasts to produce pro-inflammatory cytokines, chemokines, adhesion molecules, and matrix proteases. TNFα also stimulates the formation of osteoclasts and contributes to the blockade of regulatory T cells. All these TNFα activities fuel inflammation in the synovium, increase angiogenesis and promote cartilage and bone resorption (Alam et al. 2017).

The introduction of TNFα inhibitors has significantly improved the outcomes of rheumatoid arthritis (RA) treatment. However, up to 30% of RA patients may not respond adequately to anti-TNFα therapy (Mewar and Wilson 2011). A similar trend is observed in other chronic inflammatory diseases for which anti-TNFα regimens have been recommended (Rubbert-Roth et al. 2018). These observations suggest that the mediators other than TNFα may be driving the inflammatory reaction in some patients (Mewar and Wilson 2011; Valesini et al. 2007).

The exact mechanism by which TNFα inhibitors produce their beneficial effects is not fully understood (Alam et al. 2017). Curiously, a number of studies have revealed that TNFα inhibitors do not actually reduce the levels of soluble TNFα in blood (Eder et al. 2016a; Ohshima et al. 1999; Walters et al. 2016) and do not modulate the expression of TNFα in the synovium (Barrera et al. 2001). It is thought that binding of TNFα antibodies to the transmembrane form of TNFα on macrophages and T cells rather than simple neutralization of circulating TNFα is crucial for the effect of anti-TNFα therapy (Eder et al. 2016b). We have previously observed a more favorable clinical outcome in those patients treated with TNFα inhibitors for Crohn disease who experienced a paradoxical increase in soluble TNFα during therapy (Eder et al. 2016a). In the present study, we have aimed to determine whether serum levels of soluble TNFα change as a result of anti-TNF therapy for RA and whether they reflect disease activity and the intensity of joint pain experienced.

## Materials and methods

This was an investigator-initiated observational pilot study performed in a prospective manner. It involved 30 consecutive Caucasian patients qualified to receive anti-TNFα therapy for RA. The patients were ≥ 18 years of age and were considered eligible for therapy, according to the American–European Consensus Group (Aletaha et al. 2010) and the European League Against Rheumatism (EULAR) criteria (Smolen et al. 2017). The study protocol conformed to the ethical principles of the 1975 Declaration of Helsinki and it was approved by the Bioethics Committee of the Poznan University of Medical Sciences (No. 1067/15). Written informed consent was obtained from all participants.

The patients were treated with anti-TNFα agents (adalimumab, certolizumab, golimumab or infliximab) according to current therapeutic guidelines (Smolen et al. 2017). All patients were treated and did not respond to a prior course of standard therapy with two types of synthetic disease-modifying anti-rheumatic drugs (but were naïve to anti-TNFα therapy). Those patients who were on methotrexate continued to receive it according to the recommendations (Smolen et al. 2017). If required, corticosteroids (≤ 5 mg prednisone/day) were administered at the discretion of the attending physician, but such patients were included in the analysis, only if corticosteroids were given at the same doses for at least 4 weeks prior to and throughout the entire study period.

Patients were examined before and after 12 weeks of anti-TNFα treatment. Disease activity was evaluated by the modified disease activity score (DAS) that includes the assessment of 28 joints and of erythrocyte sedimentation rate (DAS28_ESR_) (Prevoo et al. 1995). The response to treatment was defined according to the EULAR criteria (van Gestel et al. 1996). If these criteria were not met, the patients were considered to be non-responders.

Blood samples were collected in a fasting state at the time of clinical assessment. Serum TNFα was measured with a high-sensitivity TNFα Quantikine immunoassay (R&D Systems, Minneapolis, MN, USA). All other laboratory tests were performed routinely by the hospital central laboratory.

The data are presented as medians and interquartile ranges or as percentages, as appropriate. Statistical analyses were performed using the Statistica 10.0 software (StatSoft Polska, Krakow, Poland). Normality of the data distribution was tested with the Shapiro–Wilk’s test. As the data obtained did not consistently display a normal distribution, they were analyzed with nonparametric statistics. Paired and unpaired data were analyzed with the Wilcoxon test and the Mann–Whitney test, respectively. Categorized data were analyzed with the *χ*^2^ test. The relationship between variables was analyzed with the Spearman’s rank correlation coefficient. All results were considered significant at *p* < 0.05.

## Results

**Fig. 1:**
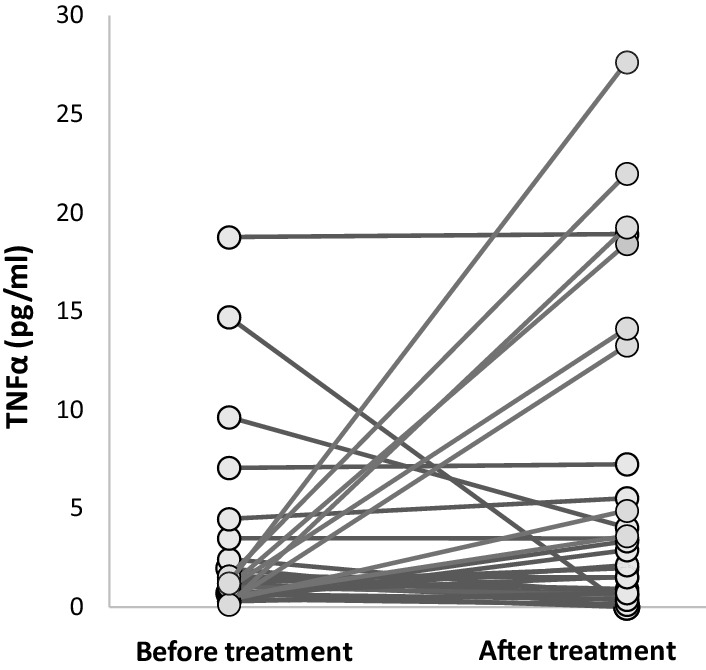
Serum TNFα levels in RA patients before and after anti-TNFα therapy (TNFα-tumor necrosis factor-alpha; *RA* rheumatoid arthritis)

Median TNF concentration in the study group rose from 1.11 (0.51–1.96) pg/mL at baseline to 2.51 (0.62–7.24) pg/mL after 12 weeks of anti-TNFα therapy. This increase was, however, not statistically significant (*p* = 0.093). We hypothesized that this was due mainly to the lack of a consistent pattern in the direction of changes across the patients (Fig. 1). While in some patients, TNFα levels fell below the detection limit, some patients experienced a manyfold increase in TNFα. A median relative change in TNFα was 13%. To assess whether the magnitude of changes in serum TNFα characterized somehow our patients, they were stratified according to whether the relative change in serum TNFα after therapy was above or below this median value.

It turned out that the patients from both subgroups did not differ in baseline clinical and biochemical characteristics and response to therapy (Table 1). As judged by clinical and biochemical criteria, 25 out of 30 patients (83%) responded well to anti-TNFα therapy and 5 patients (17%) were identified as non-responders. There was no significant difference between the groups in the distribution of responders and non-responders (4/15 vs. 1/15, *p* = 0.142). Accordingly, the responders and non-responders did not differ in terms of absolute TNFα levels and the direction of changes in TNFα over time. Consistently, there was no correlation between TNFα levels and the objective markers of disease activity (the number of swollen joints or standard laboratory markers of inflammation), equal before and after treatment.

**Table 1:** Characteristics of the study group according to the change in soluble TNFα levels during the anti-TNFα therapy in RA patients

	Patients with a relative change in serum TNFα > 13% (*n* = 15)	Patients with a relative change in serum TNFα below < 13% (*n* = 15)	*p*
TNFα (pg/mL)			
Before treatment (pg/mL)	0.60 (0.31–1.21)	1.54 (1.03–7.06)	0.059
After treatment (pg/mL)	4.89 (2.13–18.42)*^(*p* = 0.001)^	0.62 (0.13–3.47)*^(*p* = 0.001)^	<** 0**.**001**
Demographic characteristics			
Man (%)	1 (7%)	3 (25%)	0.283
Age (years)	55 (46–65)	59 (41–61)	0.803
Disease duration (years)	6 (5–9)	16 (7–18)	0.225
BMI (kg/m^2^)	23.4 (21.8–25.4)	23.6 (23.0–25.8)	0.694
*Clinical parameters of RA activity*			
DAS28			
Before treatment	5.9 (5.2–6.3)	5.3 (5.1–6.2)	0.604
After treatment	3.8 (3.1–5.1)*^(*p* = 0.001)^	3.5 (3.1–3.7)*^(*p* = 0.002)^	0.162
Non-responders (based on DAS28) [n (%)]	4 (27%)	1 (7%)	0.142
TEN28			
Before treatment	10 (7–13)	10 (9–13)	0.358
After treatment	3 (2–7)*^(*p* = 0.002)^	1 (1–2)*^(*p* = 0.001)^	**0**.**018**
SW28			
Before treatment	6 (4–7)	7 (3–9)	0.518
After treatment	1 (0–6)*^(*p* = 0.001)^	1 (0–4)*^(*p* = 0.005)^	0.801
VAS			
Before treatment	68 (60–85)	70 (60–75)	0.693
After treatment	46 (40–70)*^(*p* = 0.005)^	30 (25–49)*^(*p* = 0.005)^	0.076
*Laboratory parameters of RA activity*			
Erythrocyte sedimentation rate			
Before treatment (mm/h)	30 (24–56)	20 (10–29)	**0**.**031**
After treatment (mm/h)	20 (10–45)*^(*p* = 0.012)^	14 (7–25)	0.146
*C*-reactive protein			
Before treatment (mg/L)	7.4 (1.9–21.6)	5.2 (1.5–15.2)	0.633
After treatment (mg/L)	1.6 (0.2–9.5)*^(*p* = 0.035)^	1.0 (0.1–6.5)*^(*p* = 0.041)^	0.355
Leukocytes			
Before treatment (10^3^/µL)	9.5 (8.5–10.3)	8.3 (6.5–11.0)	0.247
After treatment (10^3^/µL)	8.5 (7.0–9.7)*^(*p* = 0.005)^	7.8 (6.6–8.6)	0.285
Neutrophils			
Before treatment (10^3^/µL)	6.2 (4.8–7.5)	4.4 (3.6–6.7)	0.849
After treatment (10^3^/µL)	4.9 (3.3–6.4)*^(*p* = 0.001)^	4.0 (3.1–5.4)	0.281
Lymphocytes			
Before treatment (10^3^/µL)	2.1 (1.7–2.2)	2.1 (1.6–3.0)	0.634
After treatment (10^3^/µL)	2.5 (2.2–2.9)*^(*p* = 0.033)^	2.4 (1.7–3.3)	0.589
Neutrophil–lymphocyte ratio			
Before treatment	2.9 (2.1–3.8)	2.1 (1.6–3.1)	0.126
After treatment	1.6 (1.3–2.4)*^(*p* = 0.002)^	1.6 (1.0–2.7)	0.844

Bold values represent statistically significant *P* values

Data presented as medians (and interquartile ranges); *p*-patients with TNFα levels increasing versus patients with TNFα levels decreasing

*DAS28* 28-joint disease activity score, *TEN28* the number of tender joints, *SW* the number of swollen joints, *VAS* visual analog scale of pain, *TNFα* tumor necrosis factor-alpha

*Before versus after

However, the patients in whom serum TNFα increased after therapy above the median value had more tender joints and tended to have higher VAS values after treatment than patients from the other group (Table 1). Consequently, the number of tender joints after the treatment correlated with absolute TNFα concentrations at this time (*r* = 0.37; *p* = 0.049) and the magnitude of changes in serum TNFα correlated with a change in the number of tender joints (*r* = − 0.48; *p* = 0.008).

## Discussion

In our study, we found no significant changes in serum TNFα levels in RA patients treated with TNFα inhibitors, despite clinical improvement. Taking into account that one of the postulated mechanisms of anti-TNFα agents’ action is the neutralization of circulating TNFα (Feldmann et al. 1997), the results of our study could be quite surprising. However, the results of our study are consistent with previous reports, in which no changes in circulating TNFα levels have been demonstrated (Barrera et al. 2001; Ohshima et al. 1999) or even higher levels of TNFα have been observed after anti-TNFα therapy (Eder et al. 2016a; Walters et al. 2016).

Probably, the decreases in soluble TNFα levels are not specific for effective anti-TNFα treatment (Barrera et al. 1993; Ohshima et al. 1999). The little is known about the alterations of cytokine levels in relation to treatment response. Targeting one of the cytokines, such as TNFα, may disrupt the cytokine network and lead to control of disease by downregulating TNFα, as well as other cytokines (Kalliolias and Ivashkiv 2016). Moreover, the efficacy of TNFα inhibitors is probably dependent on their reaction with target cells (Eder et al. 2016a, b; Kaymakcalan et al. 2009). Therefore, it seems that changes in serum TNF concentrations only to some extent reflect changes in disease progression and treatment effectiveness (Kalliolias and Ivashkiv 2016).

The present study shows that patients who experienced an increase in soluble TNFα levels had more tender joints after treatment. In this respect, the intensity of pain did not correlate with any other commonly used laboratory marker of inflammation. To the best of our knowledge, this is the first description of a possible relationship between serum TNFα concentrations and joint pain in RA patients

TNFα seems to play a significant role in the pathogenesis of chronic pain, even in diseases with no major inflammatory component. It has been shown that serum TNFα is increased in patients with fibromyalgia and non-specific low back pain (Ohgidani et al. 2017; Tsilioni et al. 2016; van den Berg et al. 2018; Wang et al. 2008). Additionally, Wang et al. (2010) demonstrated interaction between TNFα levels and pain intensity. The exact involvement of TNFα in the pathophysiology of chronic pain is not fully understood (Ohgidani et al. 2017; van den Berg et al. 2018). TNFα has been implicated in triggering mechanical nociception (Cunha et al. 1992), peripheral sensitization of nociceptors (Junger and Sorkin 2000) and central sensitization of neurons (Cuellar et al. 2004). However, the treatment with TNFα inhibitors does not lead to a significant relief of non-inflammatory pain (Molto et al. 2018).

An obvious limitation of our study is a single-center design, and the small and heterogeneous group of patients analyzed. In addition, patients received different anti-TNFα agents. Thus, it should be viewed as preliminary and be validated in an independent and larger patients’ population.

## Conclusions

Circulating TNFα levels did not decrease in RA patients treated with TNFα inhibitors, despite clinical and biochemical improvement. It is possible, that circulating TNFα is responsible for the persistence of joint pain in this group of patients.
